# Effects of Replacing Medical Zinc Oxide with Different Ratios of Inorganic: Organic Zinc or Reducing Crude Protein Diet with Mixed Feed Additives in Weaned Piglet Diets

**DOI:** 10.3390/ani11113132

**Published:** 2021-11-02

**Authors:** Han Jin Oh, Myung Hoo Kim, Min Ho Song, Ji Hwan Lee, Yong Ju Kim, Se Yeon Chang, Jae Woo An, Young Bin Go, Dong Cheol Song, Hyun Ah Cho, Min Ji Kim, Hyeun Bum Kim, Jin Ho Cho

**Affiliations:** 1 Department of Animal Sciences, Chungbuk National University, Cheongju 286-44, Korea; dhgkswls17@naver.com (H.J.O.); junenet123@naver.com (J.H.L.); xormakzm@naver.com (Y.J.K.); angella2425@naver.com (S.Y.C.); blueswing547@naver.com (J.W.A.); rhdudqls3@gmail.com (Y.B.G.); paul741@daum.net (D.C.S.); hannah0928@naver.com (H.A.C.); 2 Department of Animal Science, Pusan National University, Miryang 504-63, Korea; mhkim18@pusan.ac.kr; 3 Division of Animal and Dairy Science, Chungnam National University, Daejeon 341-34, Korea; mhsong@cnu.ac.kr; 4 Animal Nutrition and Physiology Division, National Institute of Animal Science, Rural Development Administration, Wanju 553-65, Korea; mjkim00@korea.kr; 5 Department of Animal Resource, and Science, Dankook University, Cheonan 311-16, Korea

**Keywords:** zinc oxide, alternatives, diarrhea score, zinc excretion, nutrient digestibility

## Abstract

**Simple Summary:**

Piglets frequently experience post-weaning diarrhea (PWD) due to various stress factors at the weaning stage. PWD affects the growth performance and mortality of piglets, and they face various diseases due to reduced immunity response. A high dose of zinc oxide (ZnO) is used as a feed additive to prevent diarrhea occurrence and to promote immune system development. However, most of ZnO is discharged as manure, causing soil heavy metalization, accumulation in pork, increased and antimicrobial resistance. For this reason, research to reduce zinc excretion and prevent PWD through the supplementation of low-dose zinc in feed is essential; moreover, it is essential to develop alternatives to ZnO addition. Therefore, we hypothesized that different ratios of inorganic zinc and organic zinc at 1000 mg/kg or a low-protein diet with commercial feed additives containing either essential oils, protease, and xylanase (MFA) could replace high-dose ZnO by preventing diarrhea and improving growth performance, nutrient digestibility, and gut health. We found that inorganic and organic zinc at a ratio of 500:500 mg/kg and a low-protein diet with essential oil, protease, and xylanase can be used to replace medical ZnO in weaned piglet diets.

**Abstract:**

One hundred twenty weaned piglets (9.34 ± 0.74 kg) were used in a four-week experiment to investigate the effects of replacing medical ZnO with a different ratio of inorganic and organic zinc (IZ:OZ) or a low-crude-protein diet (LP) with mixed feed additives (MFAs) in the weaned piglets’ diet. The dietary treatments included a control (CON), T1 (T1; ZnO 1000 mg/kg), T2 (IZ:OZ 850:150), T3 (IZ:OZ 700:300), T4 (IZ:OZ, 500:500), and T5 (LP with MFAs (0.1% essential oils + 0.08% protease + 0.02% xylanase)). The growth performance was decreased (*p* < 0.05) in the CON treatment compared with the T4 treatment. The diarrhea incidence was decreased (*p* < 0.05) in the T4 and the T5 treatment compared with the CON and the T1 treatments. The apparent total tract digestibility (ATTD) of nutrients were increased (*p* < 0.05) in the T4 and T5 treatments compared with the CON, T1, and T2 treatments. The T4 treatment had a higher (*p* < 0.05) ATTD of zinc than the T1, T2, and T3 treatments. The fecal microflora was improved (*p* < 0.05) in the T5 treatment compared with the CON and T3 treatments. In conclusion, IZ:OZ 500:500 could improve growth performance, nutrient digestibility, and zinc utilization while reducing diarrhea incidence in weaned piglets. Moreover, LP with MFA could replace medical ZnO.

## 1. Introduction

Post-weaning diarrhea (PWD) is caused by a variety of factors, such as the isolation of piglets from sows, the mixing of pigs in cages, adaptation to new environments, and intestinal morphological changes due to solid diet feeding [1]. These stress factors may negatively affect the immune response system and the intestinal dysfunction in weaned piglets; moreover, the undigested protein in the intestine promotes the proliferation of *Escherichia coli*, and the intestinal barrier is damaged by the enterotoxin generated by *E. coli* [2]. This condition produces enterotoxin and accretion, which are the main causes of disease outbreaks, and causes damage to the swine industry for two weeks after weaning [2]. To prevent PWD, Poulsen [3] suggested the addition of 2500 mg/kg of zinc oxide (ZnO) to piglet diets, which resulted in tremendous growth in piglet nutrition, management, breeding, and genetics [4]. The medical supplementation of the piglet diet with 2500 mg/kg ZnO promoted performance and prevented PWD for a period of two weeks after weaning. However, most ZnO is discharged as manure, causing soil heavy metalization, increased antimicrobial resistance, and accumulation in pork, which is a global problem [5,6]. Therefore, the European Union has limited the use of ZnO to 150 mg/kg and is phasing out the use in weaned piglet diets by 2022. China limits ZnO to 1600 mg/kg in weaned piglet diets [7]. However, studies on the effect of adding ZnO at concentrations of less than 2500 mg/kg are very limited and have shown conflicting results [8,9]. Thus, research to reduce zinc excretion and prevent diarrhea through the addition of low-dose zinc in feed is essential.

In our previous study, we conducted different forms of Zn such as Zn chelated with glycine and nano-particle size to replace high doses of inorganic Zn (IZ) in weaned piglet diets [10]. In our results, organic zinc (OZ) chelated with glycine had a higher utilization in weaned piglets than IZ and nanoparticle-sized zinc [10]. Moreover, many studies suggested that OZ has high stability and bioavailability, and it prevented precipitation compared to IZ [6,11,12]. It can be used as an alternative to high- dose ZnO at a lower dose.

Currently, low-protein diets, plant extracts, and enzymes have been studied as methods that do not add zinc to the diet. The recommended crude protein (CP) concentration in a piglet diet is 20–23% [13], but piglets aged 3–4 weeks lack sufficient endogenous enzymes to digest this amount of protein [14]. As the undigested protein in the intestine is the main cause of diarrhea, many researchers have investigated the effect of reducing diarrhea by using a low-protein diet (CP concentration in basal diet: 20–23% vs. low protein diet: 17.8~20.2%) [15,16,17,18]. As a feed additive, essential oil (EO) helped to improve growth performance and intestinal morphology and reduce the incidence of diarrhea in weaned piglets [19,20]. In our previous studies, a dietary supplementation of 0.1–0.2% essential oils complex in the piglet diet was shown to improve immune response and intestinal microflora [21,22]. The addition of 300–800 mg/kg protease increased the enzymatic activity and improved the apparent total digestibility of CP and amino acids, thereby improving piglet growth performance and reducing diarrhea incidence [23,24,25]. Xylanase hydrolyzes xylan in fibrin helps the proliferation of probiotic microorganisms and suppresses the proliferation of pathogenic microorganisms in the intestines, thereby improving intestinal health and enhancing immunity [26,27,28,29]. Moreover, Patráš et al. [30] reported that supplementation of 100–200 mg/kg xylanase effectively degrades non-starch polysaccharides in the upper digestive tract and improves amino acid availability in weaning piglets.

Therefore, we hypothesized that high-dose ZnO could be replaced by IZ and OZ ratios within zinc concentrations of 1000 mg/kg or by the mixed addition of EO, protease, and xylanase (MFA) in a low-protein diet (LP) by improving growth performance and reducing zinc excretion and diarrhea incidence. Thus, we conducted a study to investigate (1) the effects of replacing medical ZnO with different ratios of inorganic:organic zinc (IZ:OZ) on growth performance, diarrhea scores, nutrient digestibility, zinc utilization, blood profiles, and fecal microflora and (2) whether a low-protein diet (CP content: 0 to 2 weeks, basal diet 20.8 vs. LP diet 18.7%; 2 to 4 weeks, basal diet 18.8% vs. LP diet 16.9%) supplemented with a mixture of 0.1% EO, 0.08% protease, and 0.02% xylanase could replace medical ZnO by showing similar effects.

## 2. Materials and Methods

The experimental protocol for this study was reviewed and approved by the Institutional Animal Care and Use Committee of Chungbuk National University, Cheongju, Korea (approval #CBNUA-1530-21-01). The organic Zn was chelated with glycine (containing 27% of Zn) from Dr. Eckel Animal Nutrition GmbH & Co. KG (Anta^®^min; Niederzissen, Germany). The essential oils (Avi^®^power, containing thymol 1.4% and carvacrol 1.4%; VetAgro SpA, Reggio Emilia, Italy), xylanase (Signis^®^, AB Vista, Marlborough, UK), and protease (PT125TM, an alkaline serine endopeptidase produced by *Streptomyces* spp.; Eugene-Bio, Suwon, Korea) were mixed feed additives supported by Eugene-Bio.

### 2.1. Dietary Treatments

The dietary treatments consisted of CON (control; no additional added zinc oxide in diet), T1 (positive control; CON + 1000 mg/kg zinc oxide), T2 (CON + IZ:OZ 850:150 mg/kg), T3 (CON + IZ:OZ 700:300 mg/kg), T4 (IZ:OZ 500:500 mg/kg), and T5 (low-protein diet (LP) + mixed additives (0.1% EO + 0.08% protease + 0.02% xylanase, MFA)) in each experiment. All diets were formulated to meet or exceed the NRC (Table 1).

A total of 120 weaned piglets ((Yorkshire × Landrace) × Duroc, 28 day of age) with an initial body weight (BW) of 9.34 ± 0.74 kg were used in a four-week experiment. Pigs were blocked based on initial body weight into a complete block design. There were four pigs’ in a pen and five replicate pens per diet. All pigs were housed in an environmental controlled room (30 ± 1 °C). Each pen was equipped with a single-sided stainless self-feeder and a nipple drinker, and feed and water were freely available. Feed intake was recorded every 8:00 and 17:00, and individual pig BW was recorded at the end of weeks 0, 2, and 4 to determine the average daily gain (ADG), the average daily feed intake (ADFI), and the feed efficiency ratio (G:F). The subjective score of diarrhea was checked at 9:00 and 18:00 by the same person. We used Trckova et al.’s [31] methods to check the diarrhea score, which was assigned as follows: 0, well-formed feces; 1, normal feces; 2, sloppy feces; and 3, diarrhea. The diarrhea scores were recorded as an average daily diarrhea score for each pen and were reported as the diarrhea scores by period.

### 2.2. Chemical Analysis for Diet and Feces

We used the method of Fenton and Fenton [32] to determine the apparent total digestibility (ATTD) of gross energy (GE), dry matter (DM), and crude protein (CP). The piglet diets were mixed with 0.2% chromium oxide and fed within 10 to 14 and 24 to 28 days. Three pigs per pen were randomly selected, and fresh fecal matter was collected via rectal massage on days 14 and 28. Samples were mixed and stored in a freezer at −20 °C until analyzed. Before chemical analysis, samples were dried at 70 °C for 72 h and then finely ground to a size that could pass through a 1 mm screen. We analyzed GE, DM, and CP for all feed and fecal samples using the AOAC [33] procedure outlined. We analyzed chromium by ultraviolet absorption spectrophotometry (Shimadzu, UV-1201; Shimadzu, Kyoto, Japan) according to the chromium analysis method described by Williams et al. [34]. We used an adiabatic oxygen bomb calorimeter (Parr Instruments, Moline, IL, USA) to analyze GE in diet and feces. Diets and feces samples were wet-digested using nitric-perchloric acid and then diluted with deionized, distilled water for analyses of minerals. Concentrations of zinc were analyzed using UV absorption spectrophotometry (Shimadzu, UV-1201; Shimadzu, Kyoto, Japan).

For the blood profiles, two pigs per pen were sampled at the end of experiment. Blood samples were collected into both nonheparinized tubes and vacuum tubes containing K3EDTA (Becton, Dickinson and Co., Franklin Lakes, NJ, USA) to obtain serum and whole blood. After collection, serum samples were centrifuged (3000× *g*) for 20 min at 4 °C. The red blood cells (RBC), white blood cells (WBC), lymphocyte, monocyte, eosinophil, basophil, glucose, cholesterol, and blood urea nitrogen (BUN) levels in the whole blood were determined by using an automatic blood analyzer (ADVIA 120, Bayer, Tarrytown, NY, USA). The immunoglobulin G (IgG) and immunoglobulin M (IgM) were determined by using an automatic biochemistry blood analyzer (HITACHI 747; Hitachi, Tokyo, Japan). The zinc concentration of blood was determined according to the method described by Hill et al. [9].

### 2.3. Procedures of Microbial Shedding

Microbial analysis was immediately carried out according to the method described by Hu et al. [35]. We collected fresh fecal samples directly via rectal massage from two pigs per pen. After that, they were placed on ice and transported directly to the lab, and 1 g of a composite fecal sample from each treatment was diluted in 9 mL of 1% peptone broth (Becton, Dickinson and Co., Franklin Lakes, NJ, USA) and then homogenized. Then, viable bacteria were counted in fecal samples by placing serial 10-fold dilution on MacConkey agar plates (Difco Laboratories, Detroit, MI, USA) and lactobacilli medium III agar plates (Medium 638, DSMZ, Braunschweig, Germany) to isolate the *Escherichia coli* and *Lactobacillus*. The MacConkey agar plates were incubated for 24 h at 37 °C, and the lactobacilli medium III agar plates were then incubated for 48 h at 39 °C under anaerobic conditions. The *E. coli* and *Lactobacillus* colonies were counted immediately after removal from the incubator.

### 2.4. Statistical Analysis

Data of growth performance, nutrient digestibility, zinc utilization, and blood profiles were statistically analyzed as a randomized complete block design using the general linear model’s procedure of SAS. The diarrhea score and fecal microflora were compared with a chi-squared test, using the FREQ procedure of SAS. The pen was used as the experimental unit for growth performance, and the diarrhea score and the individual pig were used as an experimental unit for nutrient digestibility, zinc utilization, blood profiles, and fecal microflora. Orthogonal contrasts were used to compare the possible relationship about the effect of treatments: CON vs. other treatments; T1 vs. T2, T3, and T4; T5 vs. T2, T3, and T4. Variability in the data was expressed as the pooled standard error, and *p* < 0.05 was considered statistically significant.

## 3. Results

### 3.1. Growth Performance and Diarrhea Score

Pigs fed the CON and T5 diets had significantly lower (*p* < 0.05; contrast *p* < 0.05) BW than did pigs fed the T1 and T4 diets at week 2 (Table 2). The final BW was significantly decreased in the CON treatment compared with the T1, T2, T4, and T5 treatments. At 0 to 2 weeks, pigs fed with the CON and T5 diets had significantly lower (*p* < 0.05; contrast *p* < 0.01) ADG than did pigs fed the T1 and T4 diets. At 2 to 4 weeks, there was a high tendency (*p* = 0.064) for ADG in the T5 treatment compared with CON treatment.

At 0 to 2 weeks, pigs fed the CON and T1 diet had a significantly higher (*p* < 0.05; contrast *p* < 0.01) diarrhea score than did pigs fed the T4 and T5 diets. At 2 to 4 weeks, pigs fed the CON and T1 diet had a significantly higher (*p* < 0.05; contrast *p* < 0.05) diarrhea score than did pigs fed other diets. In the overall period, pigs fed the CON diet had a significantly lower (*p* < 0.05; contrast *p* < 0.05) ADG than did pigs fed the other diets. The feed efficiency was significantly decreased (*p* < 0.05; contrast *p* < 0.05) in the CON treatment compared with the T4 treatment. Pigs fed the CON and T1 diets had a significantly higher (*p* < 0.05; contrast *p* < 0.05) diarrhea score than did pigs fed the T4 and T5 diets. 

### 3.2. Nutrient Digestibility

The ATTD of DM and GE were significantly decreased (*p* < 0.05; contrast *p* < 0.05) in the CON treatment compared with the other treatments at 2 weeks (Table 3). Moreover, pigs fed the T1 and T2 diets had significantly lower (*p* < 0.05) ATTD of DM than did pigs fed the T3, T4, and T5 diets. The ATTD of CP was significantly increased (*p* < 0.05; contrast *p* < 0.05) in the T4 and T5 treatments compared with the CON, T1, and T2 treatments. Pigs fed the T4 diet had a significantly increased (*p* < 0.05; contrast *p* < 0.05) ATTD of GE than did pigs fed the CON, T1, and T2 diets. At 4 weeks, pigs fed the CON and PC diets had a significantly lower (*p* < 0.05; contrast *p* < 0.05) ATTD of DM than did pigs fed the T3 and T4 diets.

### 3.3. Zinc Utilization

Pigs fed with the PC and T1 diets had a significantly higher (*p* < 0.05; contrast *p* < 0.05) zinc intake than did pigs fed with the T3 and T4 diets at 2 weeks when the CON and T5 treatments were excluded (Table 4). Pigs fed with the PC diet had a significantly higher (*p* < 0.05; contrast *p* < 0.05) zinc excrete and a lower (*p* < 0.05; contrast *p* < 0.05) ATTD of zinc than the T2, T3, and T4 diets at 2 weeks. The zinc excrete was significantly decreased (*p* < 0.05; contrast *p* < 0.05) in the order of the T4, T3, and T2 diets according to the order of the highest OZ content at 2 weeks. The ATTD of zinc was significantly increased (*p* < 0.05; contrast *p* < 0.05) in the order of the T4, T3, T2, and T1 diets according to the order of the highest OZ content in the same period. At 4 weeks, when the CON and T5 treatments were excluded, pigs fed with the T2 diet had a significantly higher (*p* < 0.05) zinc excrete than did pigs fed with the T1, T3, and T4 diets. Pigs fed with the T4 diet had a significantly lower (*p* < 0.05) zinc excrete than did pigs fed with the T1, T2, and T3 diets. The ATTD of zinc was significantly decreased (*p* < 0.05; contrast *p* < 0.05) in the T1 treatment compared with other treatments at 4 weeks. Pigs fed with the T4 diet had a significantly higher (*p* < 0.05) ATTD of zinc than did pigs fed with other diets.

### 3.4. Blood Profiles

There was a high tendency (*p* = 0.084) for the blood concentration of monocytes in the T3 treatment compared with the CON, T1, and T2 treatments (Table 5). The BUN concentration in blood was significantly decreased (*p* < 0.05; contrast *p* < 0.05) in the T5 treatment compared with other treatments. Pigs fed with the T3 diet had a significantly higher (*p* < 0.05) blood concentration of BUN than did pigs fed with the CON and T1 diets. The zinc concentration in blood was significantly decreased (*p* < 0.05; contrast *p* < 0.05) in the CON and T4 treatments compared with the T1, T2, T3, and T4 treatments. Pigs fed with the T1 diet had a significantly higher (*p* < 0.05) zinc concentration in blood than did pigs fed with the T2 and T4 diets.

### 3.5. Fecal Microflora

The *E. coli* concentration in fecal matter was significantly decreased (*p* < 0.05; contrast *p* < 0.05) in the T3, T4, and T5 treatments compared with the CON and T1 diets (Table 6).

The *Lactobacillus* concentration in fecal matter was significantly increased (*p* < 0.05; contrast *p* < 0.05) in the T5 treatment compared with other treatments.

## 4. Discussion

The main objective of this study was to investigate alternative medical ZnO using different ratios of IZ and OZ under 1000 mg/kg or an LP diet with MFA in weaned piglet diets. Our results showed that pigs fed the T1 diet had higher growth performance than pigs fed the no-additional-ZnO-added diet. Consistent with our results, many studies have reported that supplementation with low-dose ZnO (500 to 1500 mg/kg) improved growth performance compared to no-zinc-added treatment [4,34,36]. In the present study, growth performance was improved when some of the IZ was replaced with OZ. Many researchers and our previous study found that OZ had higher bioavailability than IZ. Moreover, a low dose of OZ could replace IZ such as ZnO and ZnSO_4_ [10,11,12]. Barszcz et al. [37] reported that OZ formulations improved growth performance and increased feed intake compared to inorganic sources. Zinc chelated with glycine stimulated appetite and growth due to its role in the activity of metalloenzymes responsible for taste and the regulation of blood leptin levels [38,39]. Similar to our result, Barszcz et al. [37] reported that 460 mg/kg of zinc glycine chelate in weaning diets improved growth performance more than a conventional IZ source in the weaned periods. Wen-bin et al. [40] reported that the supplementation of weaned piglet diet with 200 mg/kg OZ improved growth performance more than high-dose ZnSO_4_. In the overall periods of our present study, the LP diet with MFA showed growth performance similar to IZ: OZ supplementation. However, in zero to two weeks, pigs fed the LP diet with MFA had lower ADG than those fed the T1 and T4 diets. Similarly, Lynegaard et al. [41] and Nyachoti et al. [42] reported that as the CP content in the diet decreased, the growth performance for two weeks after weaning decreased compared to pigs fed general feed. In two to four weeks, LP diet with MFA showed a high tendency to improve ADG compared to CON treatment. The growth performance of piglets was improved due to the high antimicrobial and immune-boosting effects of EO [20,43], and nutrient digestibility and intestinal health improved from supplementation with protease [25,44] and xylanase [30,45]. Through this result, it is thought that it will be a method that can replace high-dose ZnO in piglet diets.

PWD occurs immediately after weaning, lasts for two weeks, and causes economic loss to swine farms by decreasing growth performance and, in severe cases, causing mortality [2]. In the present study, supplementation with ZnO at 1000 mg/kg had no effect on preventing PWD and had similar diarrhea scores as pigs in the CON treatment group. However, the supplementation of weaned piglet diets with IZ:OZ at 500:500 mg/kg or the LP diet with MFA could effectively prevent PWD compared to the CON and T1 treatments. Katouli et al. [46] demonstrated that zinc in weaned piglet diets suppressed the excessive proliferation of pathogenic microorganisms (*E. coli*) by maintaining stability in the intestinal environment. As a result of this study, IZ:OZ at 500:500 mg/kg and the LP diet with MFA were considered to relieve diarrhea by improving intestinal health. In addition, Medani et al. [47] reported that a decrease in the secretion of chloride and the consequent decrease in the secretion of water in the intestines may also contribute to diarrhea. Many researchers have reported that a low-protein diet in postweaning pigs could have similar effects on preventing PWD as high-dose ZnO for pharmacological use [15,16,48]. It is considered that the addition of OZ [49] and LP diet with EO [43] and xylanase [50] had a positive effect on the immune response and prevented the incidence of diarrhea. 

In the present study, weaned piglet diets with IZ:OZ at 500:500 mg/kg and the LP diet with MFA showed higher ATTD of DM and CP. However, another study reported that there was no significant difference in nutrient digestibility when OZ was added to a weaned piglet diet or when IZ was replaced with OZ [37,51]. These findings were considered to be a result of the incidence of diarrhea and the intestinal microbial environment, whereas the LP diet with MFA improved nutrient digestibility by various actions. EO can stimulate and increase digestive enzyme secretions and gastric or intestinal motility [52,53]. The addition of protease can cleave protein chains more readily, increasing the ATTD of CP [44]. Supplementation with xylanase may involve the breakdown of cell wall non-starch polysaccharides, allowing enzymes to access nutrients, enhancing nutrient digestibility [54,55].

In our study, the zinc utilization in the zinc-added treatment groups was highest at an IZ:OZ of 500:500 mg/kg, and the zinc utilization increased as the ratio of IZ replaced with OZ increased. This was consistent with previous studies [10,56,57]. Star et al. [56] described that OZ bioavailability was increased compared to IZ owing to the nutrient transport system in the intestine, such as the amino acid or peptide transport systems. The results of this study confirmed that zinc excretion could be reduced when IZ instead of OZ was used in weaned piglet diets. However, in contrast to zinc utilization, the zinc concentrations in blood were lower in IZ: OZ at 500:500 mg/kg compared to the IZ 1000 mg/kg treatment. However, zinc supplementation resulted in higher serum zinc levels than pigs fed the no-additional-zinc-added diets (CON and T5). Many researchers reported that zinc blood concentrations were used as an indicator of zinc status and were linearly increased with zinc supplementation [58,59,60]. Our results showed that the BUN concentration was decreased in pigs fed LP with MFA diets. Consistently, previous studies also reported that the BUN concentration decreased with decreasing CP levels in the diet [15,61]. BUN is a metabolite of protein utilization and is used as an indicator to determine protein digestibility [62]. Low BUN concentrations are considered to indicate an improvement in protein utilization. 

Many researchers have reported that the dietary medical addition of high-dose ZnO to diets improved the intestinal microbiota by helping to reduce *E. coli*, a pathogenic microorganism, and aiding in the growth of beneficial bacteria [63,64]. In the present study, fecal *E. coli* concentrations, a pathogenic microorganism, were reduced in ratios of IZ:OZ from 700:300 to 500:500, which was consistent with previous studies. However, there was no difference in the *Lactobacillus* concentration between the zinc treatments (T1, T2, T3, and T4) and the CON treatment. The LP diet with MFA showed the effect of increasing the *Lactobacillus* concentrations and reducing the *E. coli* concentrations in feces. The improvement in the intestinal microbiota appeared to be due to the LP and MFA. Reducing dietary CP levels has been considered to prevent excessive protein fermentation in the large intestine and to prevent the growth of *E. coli* [14]. Peng et al. [65] demonstrated that an LP diet could improve bacterial diversity in the intestinal digesta and mucosa, which may reduce the incidence of intestinal infections from pathogenic microorganisms. The strong antibacterial and intestinal morphology improvement effects of EO have been demonstrated in many studies [19,20,65]. Through this effect of improving intestinal health, the pigs fed the LP with MFA diet in the present study showed a decreased incidence of diarrhea. Supplementation with exogenous enzymes has been shown to improve intestinal microflora due to the use of prebiotics and bioactive compounds in feedstuff [66]. Similarly, Kim et al. [67] demonstrated that the supplementation of piglet diets with multiple enzymes including protease, xylanase, phytase, amylase, and β-mannanase improved the microflora of the ileum, cecum, and feces. Supplementation with xylanase was reported to reduce the *E. coli* concentrations in the intestine using a phenolic compound cross-linked with xylan [68,69]. 

## 5. Conclusions

The addition of inorganic and organic zinc to the weaned piglet diet at a ratio of 500:500 mg/kg improved growth performance and nutrient digestibility and reduced diarrhea incidence, zinc excretion, and *E. coli* concentrations in the feces. Likewise, a low-protein diet with 0.1% essential oil, 0.08% protease, and 0.02% xylanase prevented diarrhea incidence and improved nutrient digestibility and fecal microflora with very little zinc excreted. In conclusion, inorganic and organic zinc at a ratio of 500:500 mg/kg and a low-protein diet with essential oil, protease, and xylanase can be used to replace medical ZnO in weaned piglet diets.

## Figures and Tables

**Table 1:** Compositions of the weaning diets (as-fed basis).

Items	0 to 2 Weeks		2 to 4 Weeks	
	Basal Diet	Low CP Diet	Basal Diet	Low CP Diet
Ingredient, %				
Corn	34.43	38.34	60.81	66.12
Extruded corn	15.00	15.00	5.00	5.00
Lactose	10.00	10.00	3.00	3.00
Dehulled soybean meal, 51% CP ^a^	13.50	10.00	13.00	8.07
Soy protein concentrate, 65% CP	10.00	10.00	10.00	10.00
Plasma powder	6.00	4.50	3.00	2.50
Whey	5.00	6.00	-	-
Soy oil	2.20	2.20	2.00	2.00
Monocalcium phosphate	1.26	1.26	1.15	1.15
Limestone	1.40	1.40	0.99	0.99
L-lysine-HCl, 78%	0.06	0.12	0.04	0.07
DL-methionine, 50%	0.15	0.18	0.11	0.30
Choline chloride, 25%	0.10	0.10	0.10	0.10
Vitamin premix ^b^	0.25	0.25	0.20	0.20
Trace mineral premix ^c^	0.25	0.25	0.20	0.20
Salt	0.40	0.40	0.40	0.30
Total	100.00	100.00	100.00	100.00
Calculated value ^d^				
ME, kcal/kg	3508	3503	3386	3385
CP, %	20.78	18.70	18.78	16.92
Lysine, %	1.35	1.34	1.15	1.15
Metionine, %	0.39	0.40	0.30	0.30
Ca	0.82	0.82	0.70	0.70
*p*	0.65	0.65	0.60	0.60
Analyzed value				
ME, kcal/kg	3512	3507	3402	3396
CP, %	20.92	18.76	18.82	16.94

^a^ CP, crude protein. ^b^ Provided per kg of complete diet: vitamin A, 11,025 IU; vitamin D3, 1103 IU; vitamin E, 44 IU; vitamin K, 4.4 mg; riboflavin, 8.3 mg; niacin, 50 mg; thiamine, 4 mg; d-pantothenic, 29 mg; choline, 166 mg; and vitamin B_12_, 33 μg. ^c^ Provided per kg of complete diet without Zinc: Cu (as CuSO_4_•5H_2_O), 12 mg; Mn (as MnO_2_), 8 mg; I (as KI), 0.28 mg; and Se (as Na_2_SeO_3_•5H_2_O), 0.15 mg. ^d^ Values were calculated using the National Swine Nutrition Guide (NSNG; V 2.0).

**Table 2:** Effects of replacing medical zinc oxide with different ratios of inorganic:organic zinc or reducing crude protein diet with mixed feed additives on growth performance and diarrhea score in weaned piglets.

Treatment	CON	T1	T2	T3	T4	T5	SE	*p*-Value
Inorganic:Organic Zinc	0	1000:0	850:150	700:300	500:500	LP + MFA		
Initial BW, kg	9.3	9.3	9.4	9.3	9.4	9.4	0.2	0.999
2 week BW, kg ^x, z^	13.6 ^b^	14.9 ^a^	14.6 ^ab^	14.0 ^ab^	14.9 ^a^	13.7 ^b^	0.3	0.012
Final BW, kg ^x^	21.3 ^b^	23.2 ^a^	23.0 ^a^	22.3 ^ab^	23.7 ^a^	23.0 ^a^	0.5	0.028
0 to 2 weeks								
ADG, g ^x, y, z^	330.8 ^b^	430.8 ^a^	400.0 ^ab^	361.5 ^ab^	423.1 ^a^	330.8 ^b^	20.3	0.001
ADFI, g	520.6	617.9	616.3	583.4	577.1	535.4	27.7	0.104
G:F, g/g	0.635	0.697	0.649	0.620	0.733	0.618	0.033	0.107
Diarrhea score ^1 x, y^	1.461 ^a^	1.397 ^a^	1.302 ^ab^	1.266 ^ab^	1.067 ^b^	1.154 ^b^	0.107	0.032
2 to 4 weeks								
ADG, g ^x^	550.0	592.9	600.0	592.9	628.6	664.3	25.8	0.066
ADFI, g	1228.2	1249.7	1246.3	1166.8	1271.8	1310.2	60.5	0.685
G:F, g/g	0.448	0.474	0.481	0.508	0.494	0.507	0.025	0.368
Diarrhea score ^1 x, y^	0.567 ^a^	0.505 ^a^	0.360 ^b^	0.335 ^b^	0.268 ^b^	0.367 ^b^	0.103	0.033
Overall period (0 to 4 weeks)								
ADG, g ^x^	444.4 ^b^	514.8 ^a^	503.7 ^a^	481.5 ^ab^	529.6 ^a^	503.7 ^a^	17.5	0.026
ADFI, g	887.5	945.5	943.0	885.9	937.3	937.1	37.4	0.773
G:F, g/g	0.501 ^b^	0.544 ^ab^	0.534 ^ab^	0.543 ^ab^	0.565 ^a^	0.537 ^ab^	0.020	0.255
Diarrhea score ^1 x, y^	1.101 ^a^	0.905 ^a^	0.824 ^ab^	0.802 ^ab^	0.668 ^b^	0.742 ^b^	0.063	0.012

Abbreviation: CON, no additional added zinc oxide in diet; T1, CON +1000 mg zinc oxide; T2, CON + inorganic:organic zinc 850:150 mg/kg; T3, CON + inorganic:organic zinc 700:300 mg/kg; T4, CON + inorganic:organic zinc 500:500 mg/kg; T5, low-CP diet + 0.1% essential oil + 0.08% protease + 0.02% xylanase; SE, standard error; ADG, average daily gain; ADFI, average daily feed intake; G:F, feed efficiency. ^1^ Diarrhea score was determined as follows: 0, well-formed feces; 1, normal feces; 2, sloppy feces; and 3, diarrhea ^a,b^ Means within column with different superscripts differed significantly (*p* < 0.05). ^x^ Contrast: CON vs. other treatments (*p* < 0.05); ^y^ contrast: T1 vs. T2, T3, and T4 (*p* < 0.05); ^z^ contrast: T5 vs. T2, T3, and T4 (*p* < 0.05).

**Table 3:** Effects of replacing medical zinc oxide with different ratio of inorganic:organic zinc or reducing crude protein diet with mixed feed additives on nutrient digestibility in weaned piglets.

Treatment	CON	T1	T2	T3	T4	T5	SE	*p*-Value
Inorganic:Organic Zinc	0	1000:0	850:150	700:300	500:500	LP + MFA		
2 week ATTD, %								
Dry matter ^x, y^	80.4 ^c^	81.6 ^b^	81.0 ^b^	82.9 ^a^	82.9 ^a^	83.1 ^a^	0.2	0.010
Crude protein ^x^	79.6 ^b^	79.2 ^b^	79.7 ^b^	80.2 ^ab^	81.0 ^a^	81.0 ^a^	0.3	0.001
Gross energy ^x, y^	78.8 ^c^	79.9 ^b^	79.6 ^b^	80.8 ^ab^	81.8 ^a^	80.9 ^ab^	0.3	0.013
4 week ATTD, %								
Dry matter ^x^	81.4 ^b^	81.6 ^b^	82.3 ^ab^	83.1 ^a^	83.2 ^a^	82.7 ^ab^	0.2	0.031
Crude protein ^x^	79.9	80.1	80.5	80.6	80.8	81.3	0.4	0.098
Gross energy ^x^	79.2	79.5	79.3	80.0	80.7	80.3	0.3	0.102

Abbreviation: CON, no additional added zinc oxide in diet; T1, CON +1000 mg zinc oxide; T2, CON + inorganic:organic zinc 850:150 mg/kg; T3, CON + inorganic:organic zinc 700:300 mg/kg; T4, CON + inorganic:organic zinc 500:500 mg/kg; T5, low-CP diet + 0.1% essential oil + 0.08% protease + 0.02% xylanase; SE, standard error; ATTD, apparent total tract digestibility. ^a–c^ Means within column with different superscripts differed significantly (*p* < 0.05). ^x^ Contrast: CON vs. other treatments (*p* < 0.05). ^y^ contrast: T1 vs. T2, T3, and T4 (*p* < 0.05).

**Table 4:** Effects of replacing medical zinc oxide with different ratio of inorganic:organic zinc or reducing crude protein diet with mixed feed additives on zinc utilization in weaned piglets.

Treatment	CON	T1	T2	T3	T4	T5	SE	*p*-Value
Inorganic:Organic Zinc	0	1000:0	850:150	700:300	500:500	LP + MFA		
Two weeks								
Zinc concentration, mg/kg ^x, z^	100.0 ^c^	1125.0 ^a^	1100.0 ^a^	1000.0 ^b^	1000.0 ^b^	100.0 ^c^	11.2	0.001
Average daily feed intake, g ^x, z^	520.6	617.9	616.3	583.4	577.1	535.4	27.7	0.104
Average daily zinc intake, mg ^x, z^	52.1 ^c^	695.1 ^a^	677.9 ^a^	583.4 ^b^	577.1 ^b^	53.5 ^c^	18.5	0.001
Zinc excrete, mg/kg ^x, y, z^	91.3 ^e^	1048.5 ^a^	982.3 ^b^	867.0 ^c^	827.0 ^d^	90.3 ^e^	10.0	0.001
ATTD of Zinc, % ^x, y, z^	8.7 ^c^	6.8 ^d^	10.7 ^c^	13.3 ^b^	17.3 ^a^	9.7 ^c^	0.8	0.001
Four weeks								
Zinc concentration, mg/kg ^x, z^	100.0 ^c^	1000.0 ^b^	1200.0 ^a^	1100.0 ^ab^	1050.0 ^b^	100.0 ^c^	12.4	0.001
Average daily feed intake, g ^x, z^	1228.2	1249.7	1246.3	1166.8	1271.8	1310.2	60.5	0.685
Average daily zinc intake, mg ^x, z^	122.8 ^b^	1249.7 ^a^	1495.6 ^a^	1283.5 ^a^	1335.4 ^a^	131.0 ^b^	122.7	0.001
Zinc excrete, mg/kg ^x, z^	91.0 ^d^	926.0 ^b^	1035.6 ^a^	935.0 ^b^	849.45 ^c^	89.3 ^d^	4.8	0.001
ATTD of Zinc, % ^x, z^	9.0 ^c^	7.4 ^d^	13.7 ^b^	15.0 ^b^	19.1 ^a^	10.7 ^c^	0.9	0.001

Abbreviation: CON, no additional added zinc oxide in diet; T1, CON +1000 mg zinc oxide; T2, CON + inorganic:organic zinc 850:150 mg/kg; T3, CON + inorganic:organic zinc 700:300 mg/kg; T4, CON + inorganic:organic zinc 500:500 mg/kg; T5, low-CP diet + 0.1% essential oil + 0.08% protease + 0.02% xylanase; SE, standard error; ATTD, apparent total tract digestibility. ^a–e^ Means within column with different superscripts differed significantly (*p* < 0.05). ^x^ contrast: CON vs. other treatments (*p* < 0.05). ^y^ contrast: T1 vs. T2, T3, and T4 (*p* < 0.05). ^z^ Contrast: T5 vs. T2, T3, and T4 (*p* < 0.05).

**Table 5:** Effects of replacing medical zinc oxide with different ratio of inorganic:organic zinc or reducing crude protein diet with mixed feed additives on blood profiles in weaned piglets.

Treatment	CON	T1	T2	T3	T4	T5	SE	*p*-Value
Inorganic:Organic Zinc	0	1000:0	850:150	700:300	500:500	LP + MFA		
Red blood cell, 10^6^/μL	6.90	6.86	6.97	6.56	6.44	6.24	0.27	0.328
White blood cell, 10^3^/μL	18.2	17.9	18.8	19.6	22.1	22.3	2.5	0.731
Lymphocyte, % ^z^	66.9	68.1	64.3	63.6	68.3	67.8	4.5	0.959
Monocyte, %	3.0	3.0	2.3	6.6	4.9	4.5	1.2	0.084
Eosinophil, %	0.84	0.85	1.18	1.16	1.28	0.76	0.27	0.657
Basophil, %	0.88	0.78	0.90	0.74	0.58	0.72	0.16	0.746
Immunoglobulin G, mg/dL	169.6	171.5	157.4	201.2	162.4	178.4	25.5	0.865
Cholesterol, mg/dL	80.4	80.8	85.4	74.0	87.2	85.0	5.4	0.561
Glucose, mg/dL	110.6	108.8	123.2	99.4	115.6	99.4	5.6	0.143
Blood urea nitrogen, mg/dL ^z^	6.40 ^b^	6.20 ^b^	6.60 ^ab^	7.40 ^a^	6.60 ^ab^	5.20 ^c^	0.29	0.011
Zinc, ug/dL ^x, z^	81.4 ^c^	142.3 ^a^	113.3 ^b^	109.0 ^b^	106.0 ^b^	79.7 ^c^	9.4	0.001

Abbreviation: CON, no additional added zinc oxide in diet; T1, CON +1000 mg zinc oxide; T2, CON + inorganic:organic zinc 850:150 mg/kg; T3, CON + inorganic:organic zinc 700:300 mg/kg; T4, CON + inorganic:organic zinc 500:500 mg/kg; T5, low-CP diet + 0.1% essential oil + 0.08% protease + 0.02% xylanase; SE, standard error. ^a–c^ Means within column with different superscripts differed significantly (*p* < 0.05). ^x^ contrast: CON vs. other treatments (*p* < 0.05). ^z^ Contrast: T4 vs. T1, T2, and T3 (*p* < 0.05).

**Table 6:** Effects of replacing medical zinc oxide with different ratio of inorganic:organic zinc or reducing crude protein diet with mixed feed additives on fecal microflora in weaned piglets.

Treatment	CON	T1	T2	T3	T4	T5	SE	*p*-Value
Inorganic:Organic Zinc	0	1000:0	850:150	700:300	500:500	LP + MFA		
*E. coli*, log_10_cfug^−1 x, y, z^	5.031 ^a^	4.867 ^ab^	4.609 ^ab^	4.258 ^b^	4.351 ^b^	4.326 b	0.282	0.400
*Lactobacillus*, log_10_cfug^−1 x, y, z^	7.625 ^b^	8.033 ^b^	7.737 ^b^	7.734 ^b^	7.698 ^b^	8.716 a	0.163	0.001

Abbreviation: CON, no additional added zinc oxide in diet; T1, CON +1000 mg zinc oxide; T2, CON + inorganic:organic zinc 850:150 mg/kg; T3, CON + inorganic:organic zinc 700:300 mg/kg; T4, CON + inorganic:organic zinc 500:500 mg/kg; T5, low-CP diet + 0.1% essential oil + 0.08% protease + 0.02% xylanase; SE, standard error. ^a,b^ Means within column with different superscripts differed significantly (*p* < 0.05). ^x^ Contrast: CON vs. other treatments (*p* < 0.05). ^y^ Contrast: T1 vs. T2, T3, and T4 (*p* < 0.05). ^z^ Contrast: T5 vs. T2, T3, and T4 (*p* < 0.05).

## Data Availability

Data sharing is not applicable to this article.

## References

[1] Fairbrother J.M., Nadeau É., Gyles C.L. (2005). *Escherichia coli* in postweaning diarrhea in pigs: An update on bacterial types, pathogenesis, and prevention strategies. Anim. Health Res. Rev..

[2] Rhouma M., Fairbrother J.M., Beaudry F., Letellier A. (2017). Post weaning diarrhea in pigs: Risk factors and non-colistin-based control strategies. Acta Vet. Scand..

[3] Poulsen H.D. (1998). Zinc and copper as feed additives, growth factors or unwanted environmental factors. J. Anim. Feed. Sci..

[4] Satessa G.D., Kjeldsen N.J., Mansouryar M., Hansen H.H., Bache J.K., Nielsen M.O. (2020). Effects of alternative feed additives to medicinal zinc oxide on productivity, diarrhoea incidence and gut development in weaned piglets. Animals.

[5] Jensen J., Larsen M.M., Bak J. (2016). National monitoring study in Denmark finds increased and critical levels of copper and zinc in arable soils fertilized with pig slurry. Environ. Pollut..

[6] Han X.-Y., Ma Y.-F., Lv M.-Y., Wu Z.-P., Qian L.-C. (2014). Chitosan-zinc chelate improves intestinal structure and mucosal function and decreases apoptosis in ileal mucosal epithelial cells in weaned pigs. Br. J. Nutr..

[7] Wei X., Tsai T., Howe S., Zhao J. (2021). Weaning induced gut dysfunction and nutritional interventions in nursery pigs: A partial review. Animals.

[8] Mavromichalis I., Webel D.M., Parr E.N., Baker D.H. (2001). Growth-promoting efficacy of pharmacological doses of tetrabasic zinc chloride in diets for nursery pigs. Can. J. Anim. Sci..

[9] Hill G.M., Mahan D.C., Carter S.D., Cromwell G.L., Ewan R.C., Harrold R.L., Lewis A.J., Miller P.S., Shurson G.C., Veum T.L. (2001). Effect of pharmacological concentrations of zinc oxide with or without the inclusion of an anti-bacterial agent on nursery pig performance. J. Anim. Sci..

[10] Oh H.-J., Park Y.-J., Cho J., Song M.-H., Gu B.-H., Yun W., Lee J.-H., An J.-S., Kim Y.-J., Lee J.-S. (2021). Changes in diarrhea score, nutrient digestibility, zinc utilization, intestinal immune profiles, and fecal microbiome in weaned piglets by different forms of zinc. Animals.

[11] Case C.L., Carlson M.S. (2002). Effect of feeding organic and inorganic sources of additional zinc on growth performance and zinc balance in nursery pigs. J. Anim. Sci..

[12] Schlegel P., Nys Y., Jondreville C. (2010). Zinc availability and digestive zinc solubility in piglets and broilers fed diets varying in their phytate contents, phytase activity and supplemented zinc source. Animals.

[13] National Research Council (2012). Nutrient Requirements of Swine.

[14] O’Doherty J.V., Bouwhuis M.A., Sweeney T. (2017). Novel marine polysaccharides and maternal nutrition to stimulate gut health and performance in post-weaned pigs. Anim. Prod. Sci..

[15] Heo J.M., Kim J.C., Hansen C.F., Mullan B.P., Hampson D., Pluske J. (2008). Effects of feeding low protein diets to piglets on plasma urea nitrogen, faecal ammonia nitrogen, the incidence of diarrhoea and performance after weaning. Arch. Anim. Nutr..

[16] Heo J.M., Kim J.C., Hansen C.F., Mullan B.P., Hampson D.J., Pluske J.R. (2009). Feeding a diet with decreased protein content reduces indices of protein fermentation and the incidence of postweaning diarrhea in weaned pigs challenged with an enterotoxigenic strain of *Escherichia coli*. J. Anim. Sci..

[17] Kim J.C., Heo J.M., Mullan B.P., Pluske J.R. (2011). Efficacy of a reduced protein diet on clinical expression of post-weaning diarrhoea and life-time performance after experimental challenge with an enterotoxigenic strain of *Escherichia coli*. Anim. Feed Sci. Technol..

[18] Wu Y., Jiang Z., Zheng C., Wang L., Zhu C., Yang X., Wen X., Ma X. (2015). Effects of protein sources and levels in antibiotic-free diets on diarrhea, intestinal morphology, and expression of tight junctions in weaned piglets. Anim. Nutr..

[19] Nazzaro F., Fratianni F., DE Martino L., Coppola R., De Feo V. (2013). Effect of Essential Oils on Pathogenic Bacteria. Pharmaceuticals.

[20] Rossi B., Toschi A., Piva A., Grilli E. (2020). Single components of botanicals and nature-identical compounds as a non-antibiotic strategy to ameliorate health status and improve performance in poultry and pigs. Nutr. Res. Rev..

[21] Oh H.J., Kim I.H., Song M.H., Kwak W.G., Yun W., Lee J.H., Lee C.H., Oh S.Y., Liu S., An J.S. (2019). Effects of microencapsulated complex of organic acids and essential oils on growth performance, nutrient retention, blood profiles, fecal microflora, and lean meat percentage in weaning to finishing pigs. Can. J. Anim. Sci..

[22] Liu S.D., Yun W., Lee J.H., Kwak W.G., Oh H.J., Lee C.H., Cho J.H. (2017). Effects of microencapsulated organic acids and essential oils supplementation on performance and rectal temperature in challenged weaning pigs. Anim. Prod. Sci..

[23] Angel C., Saylor W., Vieira S., Ward N. (2011). Effects of a monocomponent protease on performance and protein utilization in 7- to 22-day-old broiler chickens. Poult. Sci..

[24] Zuo J., Ling B., Long L., Li T., Lahaye L., Yang C., Feng D. (2015). Effect of dietary supplementation with protease on growth performance, nutrient digestibility, intestinal morphology, digestive enzymes and gene expression of weaned piglets. Anim. Nutr..

[25] Yu J., Yu G., Yu B., Zhang Y., He J., Zheng P., Mao X., Luo J., Huang Z., Luo Y. (2020). Dietary protease improves growth performance and nutrient digestibility in weaned piglets fed diets with different levels of soybean meal. Livest. Sci..

[26] Pollet A., Van Craeyveld V., Van de Wiele T., Verstraete W., Delcour J.A., Courtin C.M. (2012). In Vitro Fermentation of Arabinoxylan Oligosaccharides and Low Molecular Mass Arabinoxylans with Different Structural Properties from Wheat (*Triticum aestivum* L.) Bran and Psyllium (*Plantago ovata Forsk*) Seed Husk. J. Agric. Food Chem..

[27] Kiarie E., Péron A., Ru Y., Wiseman J. Xylanase improves growth performance in grower-finisher pigs fed hard and soft wheat-based diets. Proceedings of the Australasian Pig Science Association Biennial Conference.

[28] Kiarie E., Petracek R. (2015). Growth performance of nursery pigs fed pelleted wheat-based diets containing graded levels of supplemental xylanase. Anim. Prod. Sci..

[29] He X., Yu B., He J., Huang Z., Mao X., Zheng P., Luo Y., Luo J., Wang Q., Wang H. (2020). Effects of xylanase on growth performance, nutrients digestibility and intestinal health in weaned piglets. Livest. Sci..

[30] Patráš P., Nitrayová S., Brestenský M., Heger J. (2021). Effect of xylanase added to a rye-based diet on nutrient utilization in pigs. J. Microbiol. Biotechnol. Food Sci..

[31] Trckova M., Faldyna M., Alexa P., Zajacova Z.S., Gopfert E., Kumprechtova D., Auclair E., D’Inca R. (2014). The effects of live yeast Saccharomyces cerevisiae on postweaning diarrhea, immune response, and growth performance in weaned piglets1,2. J. Anim. Sci..

[32] Fenton T.W., Fenton M. (1979). An improved procedure for the determination of chromic oxide in feed and feces. Can. J. Anim. Sci..

[33] Association of Official Analytical Chemists (2007). Official Method of Analysis.

[34] Williams C.H., David D.J., Iismaa O. (1962). The determination of chromic oxide in faeces samples by atomic ab-sorption spectrophotometry. J. Agric. Sci..

[35] Hu J., Kim Y.H., Kim I.H. (2021). Effects of two bacillus strains probiotic supplement on reproduction performance, nutrient digestibility, blood profile, fecal score, excreta odor contents and fecal microflora in lactation sows, and growth performance in sucking piglets. Livest. Sci..

[36] Lei X.J., Kim I.H. (2018). Low dose of coated zinc oxide is as effective as pharmacological zinc oxide in promoting growth performance, reducing fecal scores, and improving nutrient digestibility and intestinal morphology in weaned pigs. Anim. Feed. Sci. Technol..

[37] Barszcz M., Taciak M., Tuśnio A., Čobanová K., Grešáková L.U. (2019). The effect of organic and inorganic zinc source, used in combination with potato fiber, on growth, nutrient digestibility and biochemical blood profile in growing pigs. Livest. Sci..

[38] Brandão-Neto J., Stefan V., Mendonca B., Bloise W., Castro A.V.B. (1995). The essential role of zinc in growth. Nutr. Res..

[39] Mantzoros C.S., Prasad A.S., Beck F.W., Grabowski S., Kaplan J., Adair C., Brewer G.J. (1998). Zinc may regulate serum leptin concentrations in humans. J. Am. Coll. Nutr..

[40] Wen-Bin C.H.E.N., Re-Jun F.A.N.G., Xin W.U., Cheng Z.B., Yun-Bo T.I.A.N. (2019). The effects of zinc me-thionine chelate and ZnSO_4_ on the growth performance and immune function of the weaned piglets and on IPEC-J2 cell immune function. Kafkas Üniv. Vet. Fak. Derg..

[41] Lynegaard J.C., Kjeldsen N.J., Bache J.K., Weber N.R., Hansen C.F., Nielsen J.P., Amdi C. (2021). Low protein diets without medicinal zinc oxide for weaned pigs reduced diarrhoea treatments and average daily gain. Animal.

[42] Nyachoti C.M., Omogbenigun F.O., Rademacher M., Blank G. (2006). Performance responses and indicators of gastrointestinal health in early-weaned pigs fed low-protein amino acid-supplemented diets. J. Animal Sci..

[43] Zhang J.Y., Kim Y.M., Kim I.H. (2018). Effects of dietary supplemental phytoncide instead of zinc oxide on growth performance, nutrient digestibility, blood profiles, and faecal microflora in growing pigs. J. Anim. Physiol. Anim. Nutr..

[44] Kim Y.J., Lee J.H., Kim T.H., Song M.H., Yun W., Oh H.J., Lee J.S., Kim H.B., Cho J.H. (2021). Effect of low protein diets added with protease on growth performance, nutrient digestibility of weaned piglets and growing-finishing pigs. J. Anim. Sci. Technol..

[45] Liu D., Guo S., Guo Y. (2012). Xylanase supplementation to a wheat-based diet alleviated the intestinal mucosal barrier impairment of broiler chickens challenged by Clostridium perfringens. Avian Pathol..

[46] Katouli M., Melin L., Jensen-Waern M., Wallgren P., Möllby R. (1999). The effect of zinc oxide supplementation on the stability of the intestinal flora with special reference to composition of coliforms in weaned pigs. J. Appl. Microbiol..

[47] Medani M., Bzik V.A., Rogers A., Collins D., Kennelly R., Winter D.C., Baird A.W. (2012). Zinc sulphate attenuates chloride secretion in human colonic mucosae in vitro. Eur. J. Pharmacol..

[48] Bikker P., Dirkzwager A., Fledderus J., Trevisi P., le Huërou-Luron I., Lallès J., Awati A. (2007). Dietary protein and fermentable carbohydrates contents influence growth performance and intestinal characteristics in newly weaned pigs. Livest. Sci..

[49] Zhang G., Xia T., Zhao J., Liu L., He P., Zhang S., Zhang L. (2020). Moderate tetrabasic zinc chloride supplementation improves growth performance and reduces diarrhea incidence in weaned pigs. Asian Australas. J. Anim. Sci..

[50] González-Ortiz G., Callegari M.A., Wilcock P., Melo-Duran D., Bedford M.R., Oliveira H.R., da Silva M.A., Pierozan C.R., da Silva C.A. (2020). Dietary xylanase and live yeast supplementation influence intestinal bacterial populations and growth performance of piglets fed a sorghum-based diet. Anim. Nutr..

[51] Xie Y., Zhang Q., Wang L., Wang Y., Cheng Z., Yang Z., Yang W. (2019). The effects of partially or completely substituted dietary zinc sulfate by lower levels of zinc methionine on growth performance, apparent total tract digestibility, immune function, and visceral indices in weaned piglets. Animals.

[52] Malayoğlu H.B., Baysal Ş., Misirlioğlu Z., Polat M., Yilmaz H., Turan N. (2010). Effects of oregano essential oil with or without feed enzymes on growth performance, digestive enzyme, nutrient digestibility, lipid metabolism and immune response of broilers fed on wheat–soybean meal diets. Br. Poult. Sci..

[53] Júnior C.D.S., Martins C.C.S., Dias F.T.F., Sitanaka N.Y., Ferracioli L.B., Moraes J.E., Pizzolante C.C., Budiño F.E.L., Pereira R., Tizioto P. (2020). The use of an alternative feed additive, containing benzoic acid, thymol, eugenol, and piperine, improved growth performance, nutrient and energy digestibility, and gut health in weaned piglets. J. Anim. Sci..

[54] Masey-O’Neill H.V., Singh M., Cowieson A.J. (2014). Effects of exogenous xylanase on performance, nutrient digestibility, volatile fatty acid production and digestive tract thermal profiles of broilers fed on wheat-or maize-based diet. Br. Poult. Sci..

[55] Passos A.A., Park I., Ferket P., von Heimendahl E., Kim S.W. (2015). Effect of dietary supplementation of xylanase on apparent ileal digestibility of nutrients, viscosity of digesta, and intestinal morphology of growing pigs fed corn and soybean meal based diet. Anim. Nutr..

[56] Star L., van der Klis J., Rapp C., Ward T. (2012). Bioavailability of organic and inorganic zinc sources in male broilers. Poult. Sci..

[57] Li B.T., Van Kessel A.G., Caine W.R., Huang S.X., Kirkwood R.N. (2001). Small intestinal morphology and bacterial populations in ileal digesta and feces of newly weaned pigs receiving a high dietary level of zinc oxide. Can. J. Anim. Sci..

[58] Borah S., Sarmah B., Chakravarty P., Naskar S., Dutta D., Kalita D. (2013). Effect of zinc supplementation on serum biochemicals in grower pig. J. Appl. Anim. Res..

[59] Jiao Y., Li X., Kim I.H. (2020). Changes in growth performance, nutrient digestibility, immune blood profiles, fecal microbial and fecal gas emission of growing pigs in response to zinc aspartic acid chelate. Asian Australas. J. Anim. Sci..

[60] Zhang W.-F., Tian M., Song J.-S., Chen F., Lin G., Zhang S.-H., Guan W.-T. (2021). Effect of replacing inorganic trace minerals at lower organic levels on growth performance, blood parameters, antioxidant status, immune indexes, and fecal mineral excretion in weaned piglets. Trop. Anim. Health Prod..

[61] Lee J., Kim H., Yun W., Kwak W., Lee C., Oh H., Kim D., Song M., Cho J. (2017). Effects of reducing dietary crude protein and metabolic energy in weaned piglets. S. Afr. J. Anim. Sci..

[62] Fang L.H., Jin Y.H., Do S.H., Hong J.S., Kim B.O., Han T., Kim Y.Y. (2019). Effects of dietary energy and crude protein levels on growth performance, blood profiles, and carcass traits in growing-finishing pigs. J. Anim. Sci. Technol..

[63] Yu T., Zhu C., Chen S., Gao L., Lv H., Feng R., Zhu Q., Xu J., Chen Z., Jiang Z. (2017). Dietary high zinc oxide modulates the microbiome of ileum and colon in weaned piglets. Front. Microbiol..

[64] Starke I.C., Pieper R., Neumann K., Zentek J., Vahjen W. (2014). The impact of high dietary zinc oxide on the development of the intestinal microbiota in weaned piglets. FEMS Microbiol. Ecol..

[65] Peng Y., Yu K., Mu C., Hang S., Che L., Zhu W. (2017). Progressive response of large intestinal bacterial community and fermentation to the stepwise decrease of dietary crude protein level in growing pigs. Appl. Microbiol. Biotechnol..

[66] Petry A.L., Patience J.F., Koester L.R., Huntley N.F., Bedford M.R., Schmitz-Esser S. (2021). Xylanase modulates the microbiota of ileal mucosa and digesta of pigs fed corn-based arabinoxylans likely through both a stimbiotic and prebiotic mechanism. PLoS ONE.

[67] Kim S.W., Chen H., Parnsen W. (2018). Regulatory role of amino acids in pigs fed on protein-restricted diets. Curr. Protein Pept. Sci..

[68] Arzola-Alvarez C., Hume M.E., Anderson R.C., Latham E.A., Ruiz-Barrera O., Castillo-Castillo Y., Olivas-Palacios A.L., Felix-Portillo M., Armendariz-Rivas R.L., Arzola-Rubio A. (2020). Influence of sodium chlorate, ferulic acid, and essential oils on Escherichia coli and porcine fecal microbiota. J. Anim. Sci..

[69] Mafa M.S., Malgas S., Pletschke B.I. (2021). Feruloyl esterase (FAE-1) sourced from a termite hindgut and GH10 xylanases synergy improves degradation of arabinoxylan. AMB Express.

